# Anaplerotic Therapy Using Triheptanoin in Two Brothers Suffering from Aconitase 2 Deficiency

**DOI:** 10.3390/metabo14040238

**Published:** 2024-04-20

**Authors:** Maximilian Penkl, Johannes A. Mayr, René G. Feichtinger, Ralf Reilmann, Otfried Debus, Manfred Fobker, Anja Penkl, Janine Reunert, Stephan Rust, Thorsten Marquardt

**Affiliations:** 1 Klinik für Kinder- und Jugendmedizin, Universitätsklinikum Münster, Albert-Schweizer-Campus 1, 48149 Muenster, Germanyjanine.reunert@ukmuenster.de (J.R.); stephan.rust@ukmuenster.de (S.R.); 2 Universitätsklinik für Kinder- und Jugendheilkunde, Salzburger Landeskliniken (SALK) and Paracelsus Medical University (PMU), Müllner Hauptstraße 48, 5020 Salzburg, Austria; h.mayr@salk.at (J.A.M.); r.feichtinger@salk.at (R.G.F.); 3 George-Huntington-Institut, Wilhelm-Schickard-Straße 15, 48149 Muenster, Germany; ralf.reilmann@ghi-muenster.de; 4 Clemenshospital Münster, Klinik für Kinder- und Jugendmedizin, Düesbergweg 124, 48153 Muenster, Germany; o.debus@alexianer.de; 5 Zentrale Einrichtung UKM Labor, Universitätsklinikum Münster, Albert-Schweitzer-Campus 1, 48149 Muenster, Germany; manfred.fobker@ukmuenster.de

**Keywords:** *ACO2* mutation, triheptanoin, citric acid cycle, anaplerotic therapy, case report

## Abstract

Citric acid cycle deficiencies are extremely rare due to their central role in energy metabolism. The *ACO2* gene encodes the mitochondrial isoform of aconitase (aconitase 2), the second enzyme of the citric acid cycle. Approximately 100 patients with aconitase 2 deficiency have been reported with a variety of symptoms, including intellectual disability, hypotonia, optic nerve atrophy, cortical atrophy, cerebellar atrophy, and seizures. In this study, a homozygous deletion in the *ACO2* gene in two brothers with reduced aconitase 2 activity in fibroblasts has been described with symptoms including truncal hypotonia, optic atrophy, hyperopia, astigmatism, and cerebellar atrophy. In an in vivo trial, triheptanoin was used to bypass the defective aconitase 2 and fill up the citric acid cycle. Motor abilities in both patients improved.

## 1. Introduction

**Figure 1:**
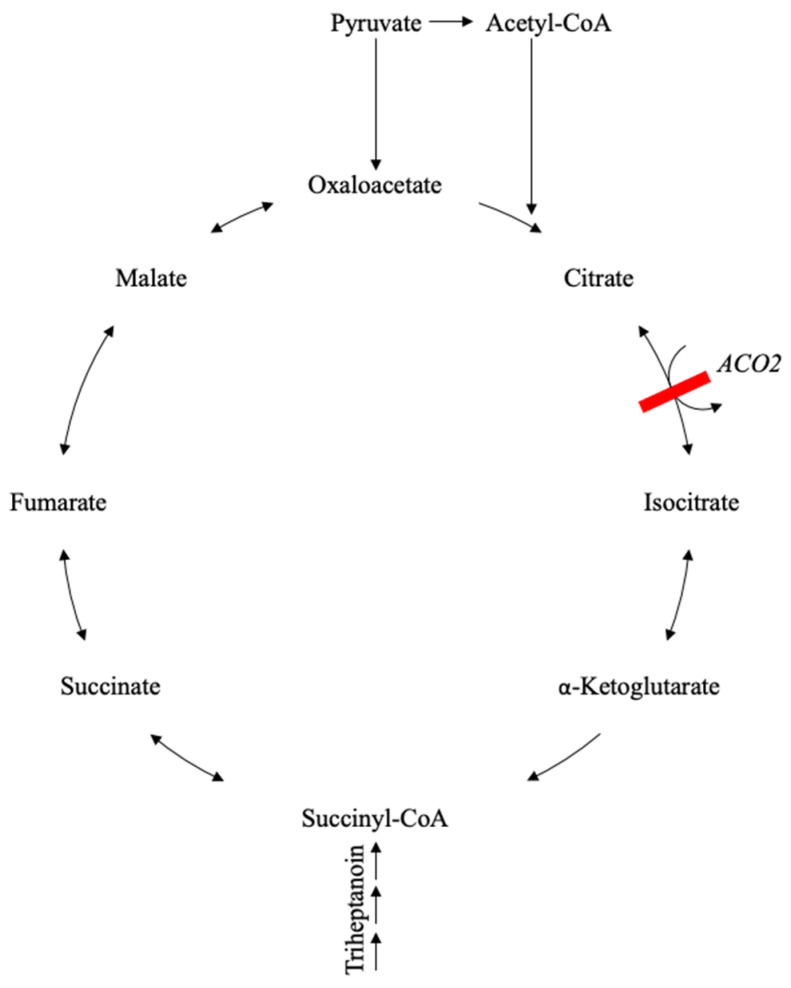
Scheme of the physiological and defective citric acid cycle in aconitase 2 deficiency with an overview of the CAC with its intermediates and bypassing Triheptanoin. The position of the aconitase 2 enzyme in CAC is illustrated as *ACO2*. Blocked pathway by defective *ACO2* marked with a red line. Triheptanoin bypasses aconitase 2 and feeds the CAC as succinyl-CoA.

The *ACO2* gene is located on chromosome 22q13.2, encoding for the mitochondrial isoform of aconitase (aconitase 2). As the second enzyme of the citric acid cycle (CAC), it is found in the mitochondrial matrix, converting citrate via cis-aconitate to isocitrate (see Figure 1). Besides being part of CAC, aconitase 2 also plays an important role in the maintenance of mitochondrial DNA (mtDNA) [1]. In *ACO2*-deficient cells, the mitochondrial respiratory capacity, the ATP turnover, the cellular ATP level [2], as well as the mtDNA copy number and mtDNA transcription, can be reduced [3]. In yeast, aconitase was found to regulate and maintain mtDNA stability. By direct binding of mtDNA, aconitase can preserve the integrity of mtDNA from the occurrence of point mutations and DNA breaks [4]. In humans, wild-type *ACO2* rescued the mtDNA depletion observed in cells from a patient with missense *ACO2* variants [1]. The maintenance of mtDNA is independent of enzymatic activity in the CAC [5]. Pathogenic variants in *ACO2* present with a neurodegenerative phenotype of variable severity [3,6], as described in over 100 cases [7]. Common symptoms are truncal hypotonia, head bobbing, and variable forms of seizures. Furthermore, patients can have ophthalmologic symptoms like strabismus, nystagmus, optic atrophy, and retinal dystrophy. Symptoms often become apparent within the first year of life [8,9]. In this study, a homozygous deletion in the *ACO2* gene in two brothers was identified. In an in vivo trial, triheptanoin, a synthetic seven-carbon fatty acid triglyceride compound that can be metabolized to succinyl-CoA, was used as an anaplerotic agent for bypassing the defective enzyme and restoring CAC function.

## 2. Materials and Methods

### 2.1. Therapy with Triheptanoin

For compassionate use, triheptanoin was provided by Ultragenyx Pharmaceutical, Novato, CA, USA. Triheptanoin was prepared with banana puree or mashed potatoes. Therapy was slowly increased to 35% of daily calories coming from triheptanoin intake.

### 2.2. Therapy Monitoring

Standard blood tests were performed, including a complete blood count, urea, creatinine, bilirubin, transaminases, and gamma-glutamyl transferase.

Organic acid excretion in the urine was repeatedly measured for metabolic therapy monitoring.

Motor abilities were quantified by Q-Motor assessments [10], and physical therapy status was assessed with the Hammersmith Functional Motor Scale for spinal muscular atrophy (HFMS) [11] or Children’s Hospital of Philadelphia Infant Test of Neuromuscular Disorders (CHOP-intended test) [12], respectively.

### 2.3. Isolation of Mitochondria from Cell Cultures

Primary human skin fibroblasts were used for the isolation of mitochondria. The medium was removed, and cell pellets were washed once with PBS. Cells were incubated for 3–5 min in a 10 mL trypsin solution for detachment. Cells were transferred quickly into FCS to inactivate trypsin. After centrifugation for 5 min at 1200–1300 units in 50 mL tubes, the supernatant was removed. Cell pellets were washed twice with 1 mL PBS. After centrifugation at 600× *g* at 4 °C for 5 min, cell pellets were weighed. Pellets were homogenized in 10 × Vol 10 mM Tris pH 7.4 with a potter 8–10 × times at maximum speed. A 1.5 M sucrose solution was immediately added to the suspension to stabilize mitochondria (volume: X = V/5; X = volume sucrose solution; V = volume resuspended cells). The suspension was mixed with a disposable plastic pipette, transferred to an Eppendorf tube, and centrifuged at 600× *g* (=1800 rpm) at 4 °C for 10 min. The supernatant, which contains the mitochondria, was centrifuged at 10,000× *g* at 4 °C for 10 min, after which the supernatant was discarded. The pellet was resuspended in 500 μL SEKT (250 mM sucrose, 2 mM EGTA, 40 mM KCl, and 20 mM Tris. pH 7.4) buffer. After centrifugation of the solution at 10,000× *g* at 4 °C for 10 min, the supernatant was discarded, and the pellet was resuspended in 1 vol of SEKT buffer (if the initial cell pellet has 50 mg, the final volume is 50 µL). The aliquoted pellets were subsequently stored at −70 °C.

### 2.4. Western Blotting

Isolated mitochondria from primary human skin fibroblasts were used for Western blotting. In brief, mitochondrial lysates were loaded onto a 10% SDS-PAGE gel. Electrophoresis was performed at 150 V for 35 min. Proteins were blotted onto nitrocellulose membranes (Transblot Turbo Pack; Biorad, Hercules, CA, USA) with the Transblot Turbo System from Biorad. Blocking was carried out using the Western blocking reagent (Roche, Basel, Switzerland) for 30 min at room temperature. Membranes were incubated with the following primary antibodies: mouse monoclonal anti-*ACO2* antibody overnight at 4 °C (Abcam, Cambridge, UK; ab110321) and mouse monoclonal anti-VDAC1 antibody 1 h at room temperature (Abcam, ab14734). The secondary antibody from the DAKO Envision kit was used. The Lumi-Light Western blotting substrate (Roche) was used for development.

### 2.5. Aconitase Activity

In order to determine aconitase activity, a commercially available kit was used (BIOXYTECH Aconitase-340; catalog number 21041). A total of 50 µg of isolated mitochondria from human skin fibroblasts were diluted in assay buffer to a final volume of 250 µL. Subsequently, 250 µL substrate, 250 µL enzyme, and 250 µL NADP were added (final reaction volume: 1 mL). Substrate, enzyme, and NADP are included in the aconitase kit. The solution was sonified for 20 s at low energy. After 15 min of preincubation at 37 °C, the change in absorbance was measured at 340 nm.

### 2.6. Monitoring of Plasma C7 Fatty Acids

**Figure 2:**
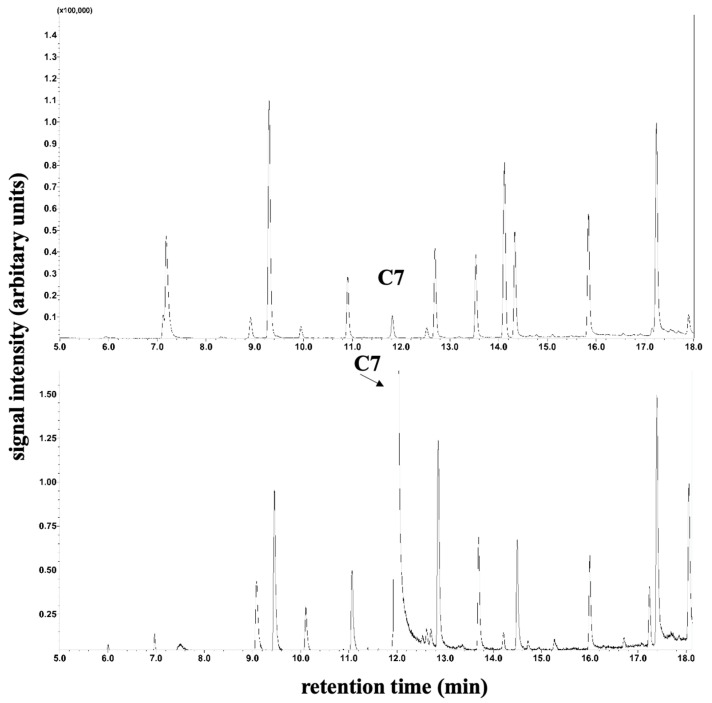
Determination of plasma C7 fatty acids for patient 1. Section of peak intensity chromatogram of plasma medium-chain free fatty acids by gas chromatography–single quadrupole mass spectrometry. The top image shows plasma C7 fatty acids prior to therapy, and the bottom image shows measurements during therapy.

**Figure 3:**
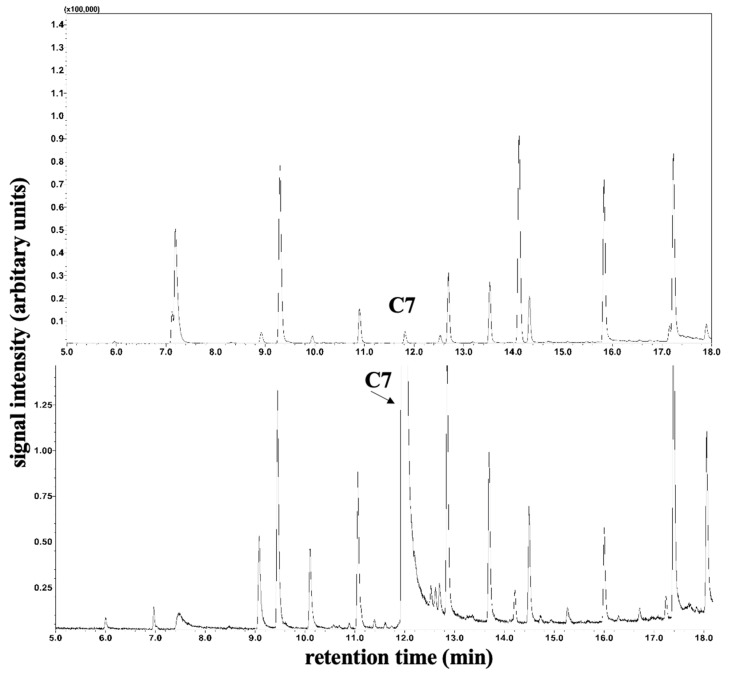
Determination of plasma C7 fatty acids for patient 2. Section of peak intensity chromatogram of plasma medium-chain free fatty acids by gas chromatography–single quadrupole mass spectrometry. The top image shows plasma C7 fatty acids prior to therapy, and the bottom image shows measurements during therapy.

The determination of plasma C8 and C10 fatty acid levels was performed by gas chromatography–mass spectrometry. For this purpose, 100 µL serum was mixed with 10 µL internal standard (20 µg/mL D3-Octanoic acid in methanol), 20 µL 37% HCl/H2O 1:1 (*v*/*v*), 200 µL H2O, 100 µL Methyl-t-butyl ether, and 50 µL n-hexane in a 0.5 mL Eppendorf reaction vessel and centrifuged (3 min, 10,000× *g* rpm). A total of 50 µL of the supernatant was transferred to an autosampler vial with a conical insert, and 1 µL was loaded in splitless mode onto a GC–MS (QP2010 Ultra; Shimadzu, Duisburg, Germany) equipped with a PTV injector and a capillary column (Stabilwax-DA 15 m length, 0.25 mm internal diameter, and 0.25 µm film thickness; Restek GmbH, Bad Homburg, Germany) and an autosampler AS AOC-20s. Measuring time: 58.7 min; injector temperature: 70 °C; column oven temperature program isothermal: 2 min at 40 °C; gradient 12 °C/min to 240 °C; isothermal: 40 min at 240 °C. The carrier gas was helium with a constant flow of 1 mL/min. The transfer line to the ion source was held at 260 °C. The MS detector was set to selected ion monitoring (SIM) mode as *m*/*z* 60 (C8/C10) and *m*/*z* 63 (D3-Octanoic acid, internal standard). An example of a base peak intensity chromatogram of free fatty acids is shown in Figure 2 and Figure 3. LabSolutions software (Version 2.72; Shimadzu, Duisburg, Germany) was used for GC–MS data acquisition and processing.

## 3. Results

### 3.1. Clinical Report

The patients were born to a consanguineous couple from Yemen. The firstborn boy (patient 1) was born by cesarean section after an uneventful pregnancy with a birth weight of 2840 g, a length of 49 cm, and an occipitofrontal circumference of 35 cm. Apgar scores were 10/10/10.

Hexadactyly was present on both hands and was removed surgically on the third day of life.

Up until a febrile infection at the age of eight months, the development was normal. Simultaneously with the infection, the child became extraordinarily floppy, was unable to stabilize his trunk, and his head showed a head tilt. His targeted movements were atactic, and at rest, his head and body were slightly shaking. Reflexes could not be elicited clearly. His eyes exhibited a slow horizontal nystagmus. Wakefulness and interaction were not disturbed. Seizures were excluded by EEG–liquor, and MRI analysis was normal.

At the age of one year, a motor development slowdown, as well as truncal hypotonia, had progressed—even under physiotherapy. To objectify physical therapy status, 35/66 points on the Hammersmith Functional Motor Scale for spinal muscular atrophy (HFMS) [11] were calculated. In the course, infantile esotropia, downbeat nystagmus, optic atrophy, hyperopia, astigmatism, and cerebellar atrophy were diagnosed. While growing up, poor pronunciation was noticed.

At the age of eight years, a genetic analysis was performed due to the fact that the younger brother (patient 2) displayed similar symptoms.

Patient 2 was born via a planned cesarean section after an uneventful pregnancy with a birth weight of 2810 g, a length of 47 cm, and an occipitofrontal circumference of 34.8 cm. Apgar scores were 10/10/10. Development was normal until the patient was 1 month old. After a febrile infection, the boy displayed the same symptoms as the older brother. In order to objectively measure the boys’ physical therapy status, the Children’s Hospital of Philadelphia Infant Test of Neuromuscular Disorders (CHOP-intended test) [12] was chosen. Here, 44/64 points were calculated.

### 3.2. Genetic Analysis

Whole-exome sequencing identified the homozygous variant c.2335_2340delCAACAG, p.Gln779_Gln780del, i.e., deletion of the last two amino acids of the protein, for both brothers in *ACO2* (NM_001098.3). This variant was predicted to be pathogenetic by in silico simulation [13,14,15,16].

### 3.3. Fibroblasts Analysis

**Figure 4:**
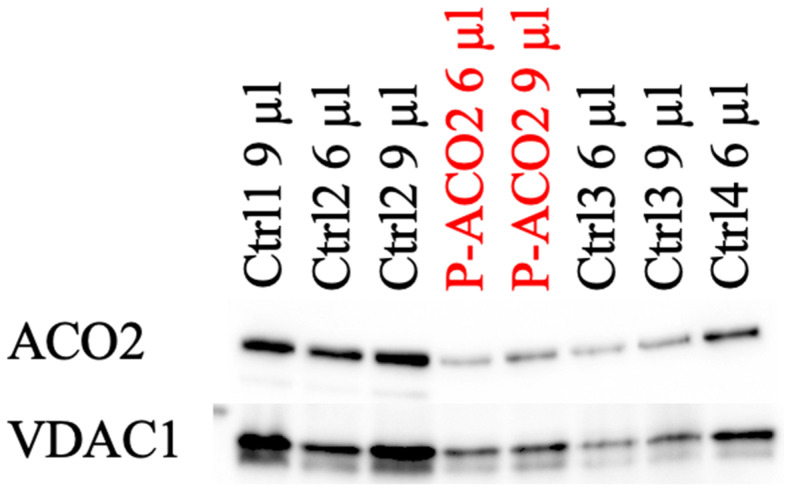
Western blot analysis of the *ACO2* levels of the affected individual and four controls.

**Figure 5:**
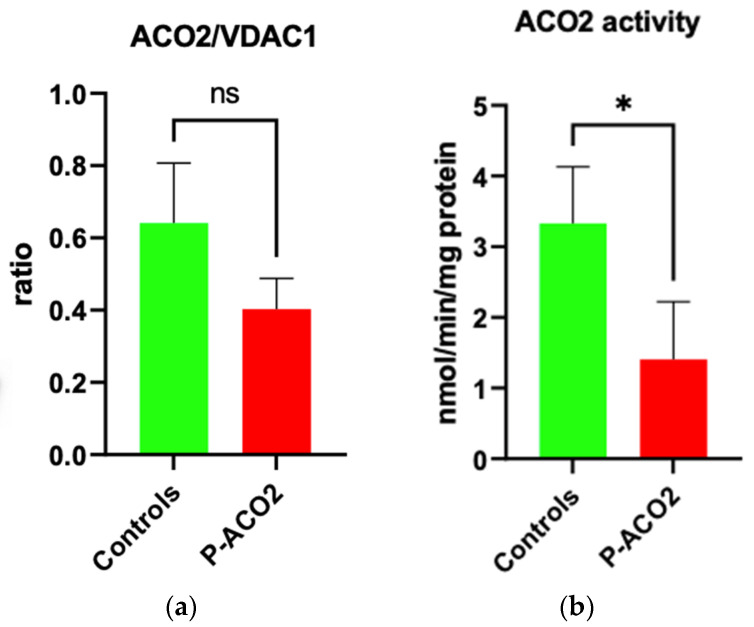
Determination of *ACO2* activity in the affected individual and controls. (**a**) Densitometric analysis of the *ACO2/VDAC1* ratio of the Western blot results. (**b**) Enzymatic activity of *ACO2* in the affected individual and controls. VDAC1 was used as a mitochondrial loading control. A non-parametric Mann–Whitney U test was used for statistical analysis. The enzymatic measurement was repeated twice with the same controls used in Figure 3. For both experiments, isolated mitochondria from human primary skin fibroblasts were used. For the enzymatic determination, 50 µg mitochondria per 1 mL assay volume were taken. Values present mean ± SD. (**a**): *p* = 0.28; (**b**): * *p* = 0.0238.

Skin fibroblasts were taken and cultured [17] from the older brother in order to quantify the residual enzyme activity and protein level of aconitase 2, with four controls also being measured for comparison purposes. In Western blot, protein levels were reduced (see Figure 4). As seen in Figure 5, aconitase 2 activity was significantly reduced to 40% compared to the four controls.

### 3.4. Therapy with Triheptanoin

Triheptanoin was started with a low dose and increased to a daily amount of 35% triheptanoin when the caloric intake was reached. During therapy, nausea and vomiting occurred, leading partially to the rejection of the prepared food. Nevertheless, the treatment could still be continued.

### 3.5. Motor Ability Analysis

**Figure 6:**
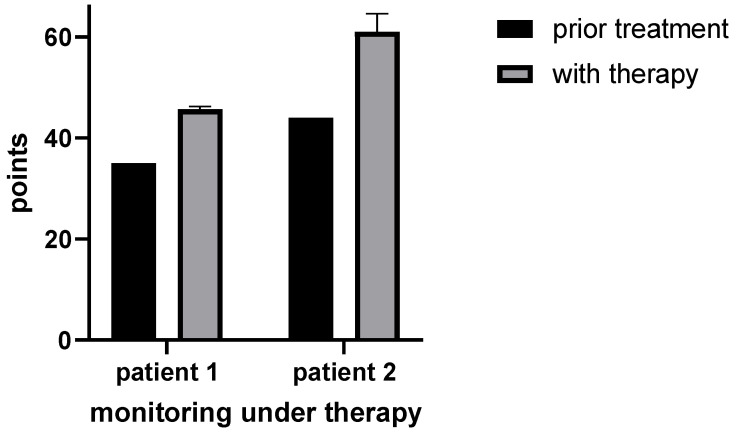
Monitoring of motor ability with HFMS for patient 1 and CHOP for patient 2. Patient 1: Sum of calculated points in HFMS before (*n* = 1) and during therapy (*n* = 3). Patient 2: Sum of calculated points in CHOP before (*n* = 1) and during therapy (*n* = 3).

**Figure 7:**
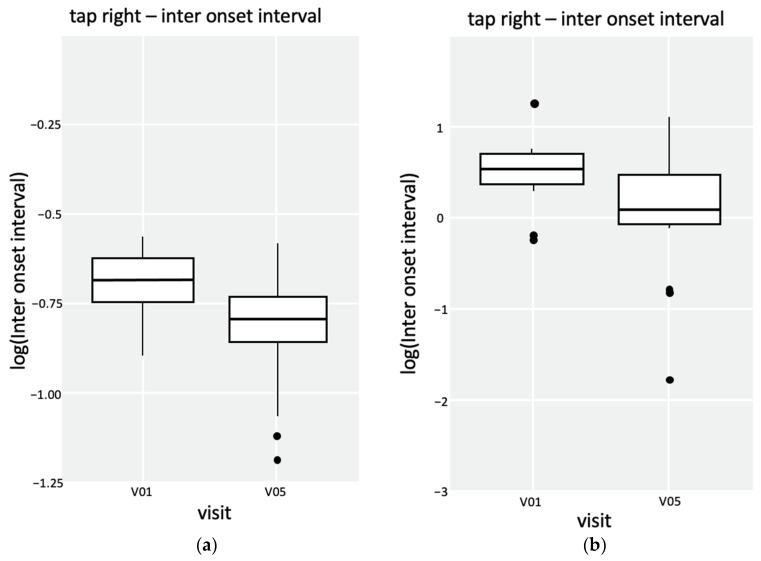
Q-Motor analysis of patient 1 with box plots of speeded tapping tests of the right hand and the left foot. (**a**) Speeded tapping performance of the right hand. The *p*-value in the Wilcoxon rank sum test is 9.813^−9^. (**b**) Speeded tapping performance of the left foot. The *p*-value in the Wilcoxon rank sum test is 0.003691.

Depending on the patient’s age, either HFMS combined with Q-Motor assessments [10] or a CHOP-intended test was used for the monitoring of motor ability analysis. Prior to therapy, patient 1 scored 35/66 points at HFMS. After two months of therapy, the boy improved to 46/66 points (HFMS) and was able to maintain this level during therapy. Additionally to HFMS, Q-Motor assessments were used for patient 1. The data suggested that speeded tapping performance had improved. With a *p*-value < 0.05 in the Wilcoxon rank-sum test, the data are significant for the right hand and left foot (see Figure 6 and Figure 7). For patient 2, 44/64 points in the CHOP-intended test were calculated prior to the therapy. After two months of therapy, the boy reached 60/64 points and 56/64 points after six months of therapy (see Figure 6).

### 3.6. Organic Metabolites

**Figure 8:**
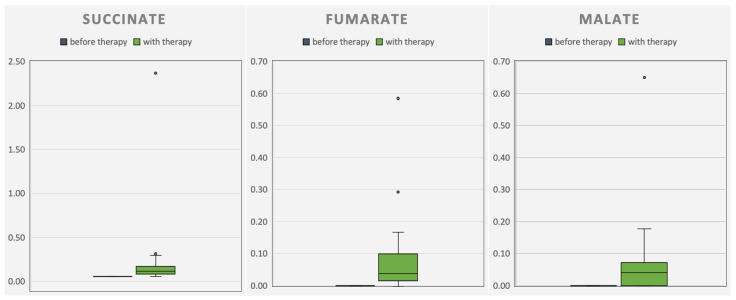
Progression of the measured excretory organic acids succinate, malate, and fumarate in the urine of patient 1. The organic acids fumarate and malate before therapy (blue) were not detectable (*n* = 5). With therapy (green), a higher excretion of the organic acids was detected (*n* = 38).

**Figure 9:**
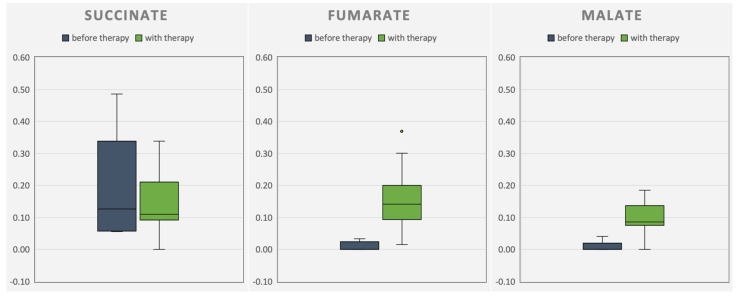
Progression of the measured excretory organic acids succinate, malate, and fumarate in the urine of patient 2. Five samples before therapy (blue; *n* = 5). With therapy (green), a higher excretion of the organic acids fumarate and malate was detected (*n* = 38).

Concurrent with the improvements in HFMS or CHOP-intended tests, respectively, an increase in CAC intermediates in urine organic acid analysis was observed during the therapy phase (see Figure 8 and Figure 9). At the same time, the tremor of patient 1 disappeared, accompanied by improved pronunciation. Improvements in patient 2 were reduced squinting under therapy. On days with high energy demands (e.g., when affected by infection), patient 2 presented with a whole-body tremor again.

### 3.7. C7 Fatty Acids

According to a modified method of Bartolucci et al. [18], the plasma C7 fatty acids were elevated during therapy compared to the control measured for comparison purposes (see Figure 2 and Figure 3) by gas chromatography–mass spectrometry.

### 3.8. Follow-Up

After two years of therapy, the patients’ parents decided to discontinue triheptanoin therapy because the success in daily life fell below the parents’ expectations. During the follow-up six months after stopping the therapy, patient 1’s pronunciation had deteriorated, and there was also again a marked reduction in body tension without therapy.

## 4. Discussion

The present study provides the first description of triheptanoin in the treatment of aconitase 2 deficiency.

Mutations in *ACO2* have been described in association with early fatal or neurodegenerative diseases. Clinical symptoms range from mildly affected patients with isolated optic atrophy to severely affected patients, including intellectual disability and seizures in early childhood. Also, hypotonia, optic nerve atrophy, cortical atrophy, and cerebellar atrophy are possibly occurring [3,7,9].

Previous studies suggested a correlation between the severity of clinical symptoms and aconitase 2 activity. In these studies, aconitase 2 activity ranged from over 60% to 5% in skin fibroblasts from patients with homozygous or compound heterozygous mutations [1,9,19]. In the case of reduced enzyme activities, a certain tissue specificity must always be considered. The degree of reduction is possibly more pronounced in other, more energy-demanding tissues than in skin fibroblasts.

Patients with higher aconitase 2 activity had a milder clinical phenotype. The same studies could show that aconitase 2 activity did not correlate with aconitase 2 protein levels [1,9,19].

While the protein level of patient 1 decreased (not significantly), there was a reduction in enzyme activity to 40%. Compared to the patients described by Metodiev et al., the phenotype is milder compared to 5% remaining activity but more severe compared to 60% remaining enzyme activity [19]. Additionally, previous investigations found a decreased mitochondrial respiratory capacity, ATP turnover, cellular ATP level, and mtDNA copy number in *ACO2*-deficient cells [1,2,3].

Assuming that aconitase deficiency results in insufficient energy provision, bypassing the defective aconitase 2 and increasing the mitochondrial respiratory capacity, ATP turnover, and cellular ATP level was the treatment approach. Due to the fact that aconitase 2 converts citrate to isocitrate, which cannot proceed normally in *ACO2*-deficient cells, direct isocitrate supplementation might be beneficial. Indeed, Chen et al. found an increase in cellular ATP levels in *ACO2*-deficient cells compared to controls after isocitrate supplementation in vitro [2]. Preparation of isocitrate is possible, e.g., by using yeast fermentation of sunflower oil [20]. The production of large quantities of isocitrate by this method is costly. Due to a lack of information about isocitrate uptake and metabolism in vivo, an alternative was sought. Physiologically, in CAC, isocitrate is converted to α-ketoglutarate, the next potential target for anaplerotic therapy. Direct α-ketoglutarate supplementation is possible with ornithine α-ketoglutarate; indirect supplementation is possible with a precursor of α-ketoglutarate. Longo et al. tested both ornithine α-ketoglutarate and glutamine as a precursor. They found neither ornithine α-ketoglutarate nor glutamine could increase downstream intermediates of CAC [21].

It was decided to start a trial with triheptanoin for compassionate use because it was described as having good potential in metabolic and neurodegenerative diseases [22,23,24,25]. Additionally, the application is easy to handle in homecare and has few side effects at the right dose. Triheptanoin is a synthetic seven-carbon fatty acid triglyceride compound where three C7 fatty acids (heptanoate) are esterified to one glycerol. Triheptanoin comes as an oil, which, when ingested, probably disseminates like other medium-chain triglycerides (MCT) fatty acids [26] via the venous system, mainly to the liver [27,28,29]. At the target cells, no active transport is required. Heptanoate diffuses through the cell and mitochondrial membrane to enter ß-oxidation by the medium-chain fatty-acid oxidase enzyme. Besides liver cells, heptanoate can be oxidized in mitochondria by most tissues [24].

Through two cycles of ß-oxidation, one molecule of heptanoate is degraded into two molecules of acetyl-CoA and one propionyl-CoA. While acetyl-CoA can enter CAC directly via citrate synthase, propionyl-CoA can be carboxylated and converted to succinyl-CoA via methylmalonyl-CoA by propionyl-CoA carboxylase, methylmalonyl-CoA epimerase, and methylmalonyl-CoA mutase. Formed succinyl-CoA can then enter CAC as an intermediate and might be used for gluconeogenetic reactions [23,27].

A second opportunity for heptanoate metabolization is to undergo just one cycle of ß-oxidation. The result is one acetyl-CoA and one remaining pentanoyl-CoA. Then, pentanoyl-CoA can be used to generate the C5-ketone bodies β-ketopentanoate and β-hydroxypentanoate. The C5-ketone bodies can cross the blood-brain barrier in order to support the brain. Subsequently, the ketone bodies are converted by the peripheral tissues to acetyl-CoA and anaplerotic propionyl-CoA [23,27]. As shown in Figure 1, triheptanoin feeds the CAC behind aconitase 2 as succinyl-CoA.

Due to the rarity of aconitase 2 deficiency, there is, to date, no specific monitoring test. For the presented patients, problems with motor mobility were the most noticeable and limiting restrictions in their daily routine. To achieve a general overview of treatment response, motor ability with Q-Motor assessments, HFMS, and CHOP-intended tests were monitored. Special monitoring with regard to, e.g., retinal dystrophy was performed in standard care and was not considered within the framework of this investigation paper.

Q-Motor assessments are usually used for neurodegenerative Huntington’s disease, which presents with a variety of motor symptoms. Active cooperation of the patient is required in order to perform the tests; therefore, only patient 1 was able to take part in Q-Motor assessments.

Q-Motor assessments provide standardized measurements at different time points to avoid bias induced by examiners or study side effects. Data analysis is free from bias through blinded and automated analysis [10]. For patient 1, there was an indication of improved tapping performance in the right hand and the left foot (see Figure 7). Simultaneously with Q-Motor assessments, physical therapy status with the Hammersmith Functional Motor Scale for spinal muscular atrophy (HFMS) for patient 1 and the CHOP-intended test for patient 2 were measured. HFMS is a functional motor scale for children with spinal muscular atrophy, in particular for those with limited mobility, to provide objective information on motor ability and clinical progression [11].

The CHOP-intended test is also used to evaluate the motor skills of patients with spinal muscular atrophy, but even for infants [12].

During therapy with triheptanoin, motor abilities increased in both patients. The motor abilities of patient 1 increased from 35/66 to 46/66 points (HFMS) after two months of therapy. Patient 2 improved from 44/64 points to 56/64 points (CHOP-intended test). Even though these motor ability tests were not developed specifically for aconitase 2 deficiency and may not accurately capture all aspects of the described symptoms, this work shows that at least some of the symptomatic complex of aconitase 2 deficiency is caused by insufficient energy provision.

## 5. Conclusions

Pathogenic variants in *ACO2* are rare but can be found by whole-exome sequencing. When *ACO2* activity is reduced, anaplerotic triheptanoin might be beneficial. After ingestion, systemic uptake of triheptanoin has been proven by gas chromatography–mass spectrometry of the patients’ plasma. By measuring the elevated levels of the excretory organic acids succinate, malate, and fumarate in the urine (in CAC behind defective *ACO2*), anaplerosis and further metabolization could be shown. Improved motor abilities, verified by Q-Motor assessments, CHOP-intended tests, and HFMS, are attributable to anaplerotic triheptanoin.

## Data Availability

The original contributions presented in the study are included in the article, further inquiries can be directed to the corresponding authors.
